# Supplementary material for: Determining the vitamin D supplementation duration to reach an adequate or optimal vitamin D status and its effect on blood lipid profiles: a longitudinal study
Source: J Health Popul Nutr. 2024 Jun 12;43:81. doi: 10.1186/s41043-024-00576-6 (PMC11170904; doi:10.1186/s41043-024-00576-6)
Supplement: Supplementary file 1 — Supplementary Material 1 [file 41043_2024_576_MOESM1_ESM.docx]

**Determining the vitamin D supplementation duration to reach an adequate or optimal vitamin D status and its effect on blood lipid profiles: A longitudinal study**

**Sakineh Nouri Saeidlou^1,^ Davoud Vahabzadeh*^2^, Fozieh Karimi^3^, Fariba Babaei^4^**^su^

^1^Food and Beverages Safety Research Center, Urmia University of Medical Sciences, Urmia, Iran

^2*^ Non-Communicable Disease Research Center, Ilam University of Medical Sciences, Ilam, Iran

^3^ Midwifery Department, Ilam University of Medical Sciences, Ilam, Iran

^4^ Department of Health Affairs, Urmia University of Medical Sciences, Urmia, Iran

Corresponding author: Davoud Vahabzadeh

Address: Associate Professor, Non-Communicable Disease Research Center, Ilam University of Medical Sciences, Ilam, Iran

Email: vahabzadeh-d@medilam.ac.ir. Phone No: +988432227126; Fax: +988432227120

**Abstract**

**Background**: Recently, Serum vitamin D (Vit. D) levels evaluation and the use of Vit. D supplements have increased substantially. There is no specific guideline for the duration of Vit. D supplementation, so yet Vit. D supplementation duration has remained a critical and controversial issue. This study aimed to determine the vit. D supplementation duration to reach an adequate or optimal Vit. D status and its effect on lipid profile.

Methods: In this longitudinal study, 345 women with different status of Vit. D levels were enrolled and followed up for one year. Eligible participants received 50000 IU Vit. D_3_ (cholecalciferol) once a month for 12 consecutive months. The serum Vit. D levels and lipid profiles were measured at baseline, 3rd, 6th, and 12th months after the intervention. Participants were categorized based on Vit. D level at baseline into deficiency (<20 ng/mL), inadequate (20-30 ng/mL), and adequate (>30 ng/mL) groups, and the data were compared at different times between the three groups.

Results: Three deficiency (n=73), inadequate (n=138) and adequate (n=134) groups of participants were followed. In all participants the average amount of Vit. D level changes were 8 ng/mL after one year of supplementation. The mean changes of serum Vit. D level in 6th and 12th months vs. 3th month was as below: In deficiency group: 4.08±0.85 and 10.01±1.02 ng/mL; (p<0.001), in inadequate group: 3.07±0.59 and 7.26±0.78 ng/mL; (p=0.001) and in adequate group: 2.02±0.88 and 6.44±1.005 ng/ml; (p=0.001). Lipid profiles were improved in three groups. So, the mean changes of lipid profiles at the end of the study comparing the baseline were, -5.86±2.09, -7.22±1.43 and -6.17±1.72 (mg/dl) for LDL (p<0.05); -12.24±3.08, -13.64±3.21 and -17.81±2.94 (mg/dl) for cholesterol (p<0.05) in deficiency, inadequate and adequate groups, respectively. For triglyceride, the mean changes were -13.24±5.78 and -15.85±7.49 (mg/dl) in deficiency and adequate groups, respectively (p<0.05). Although the triglyceride decreased in the inadequate group at the end of the study but this difference was not significant (p=0.67).

**Conclusion:** Taking of 50000 IU Vit. D 3 monthly for 12 months resulted in reaching its level to adequate level in both deficiency and insufficient groups; however, in the adequate group its level did not reach above than 50 ng/mL. Therefore, 50000 IU Vit. D_3_ supplementation monthly for one year can have beneficial effects on lipid profiles and there is no risk of toxicity in healthy women.

**Keywords**: vitamin D, supplementation duration, lipid profiles, adequate levels of vitamin D

**Introduction**

Vit. D is a fat-soluble vitamin and it plays an important role in the homeostasis of calcium and bone metabolism (1-4). The prevalence of Vit. D deficiency is a global public health problem 1, (5). About one billion people worldwide have Vit. D deficiency, while 50% of the population has Vit. D insufficiency (1). Studies have shown that Vit. D deficiency is associated with many chronic diseases such as cancers, diabetes mellitus, cardiovascular diseases, infections, lipid abnormalities, and autoimmune diseases (6-9).

The source of Vit. D can be sunlight exposure, diet, and supplementation (4). According to the current scientific data, the adequate Vit. D concentration is associated with a wide variety of health conditions (10). Vit. D supplementation can be a safe, economical, and widely available method (2). Recently, serum Vit. D levels evaluation and the use of Vit. D supplements have increased substantially (5), But there is still no general agreement on determining the Vit. D levels and the role of Vit. D supplementation; so, the optimal Vit. D dose and tacking its changes is a subject of debate (11). Currently, there is uncertainty regarding the duration of Vit. D supplementation for reaching adequate or optimal levels and the use of supplements of Vit. D is controversial (12).

Serum 25-hydroxy Vit. D (25OHD) is the most commonly used marker of Vit. D status, because it has a relatively long half-life (13). The definition for Vit. D deficiency and the level of Vit. D requirement for different age groups is yet controversial (12,14,15). The current cut-offs for defining deficiency/insufficiency/adequacy level of Vit. D is yet challengeable (16). Almost the majority of current guidelines state that Vit. D 33 level <20 ng/mL should be considered deficiency, 20-30 ng/mL as insufficiency, and >30 ng/mL as sufficiency (17,18). Scientific evidence shows that, Vit. D deficiency may be associated with many chronic diseases; on the other hand, its level above than 100 ng/ml can lead to some other disorders that are related to its toxicity (19).

In several studies the effect of different durations of supplementation with 50000 IU Vit. D has been studied with different health outcomes (20-23). However determining the Vit. D supplementation duration is still controversial. Since Vit. D deficiency can be the cause of many health-related diseases, also its toxicity can cause many complications, including increased calcification of soft tissues, cardiovascular complications, etc.

One of the proposed mechanisms for the association between vitamin D and some metabolic disorders is its postulated effect on blood lipid profiles. About 12–15% of studies in one meta-analysis have pointed to an inverse relationship between TG (triglyceride) and serum levels of 25 (OH) D (16). Also, some studies have shown a positive correlation between vitamin D status and HDL (high-density lipoprotein) while reporting the inverse association of vitamin D status with LDL (low-density lipoprotein) (16-19). Hypovitaminosis D was shown to be associated not only with lowered insulin secretion and sensitivity but also with adverse effects on TG, total cholesterol, and LDL-cholesterol and HDL-cholesterol concentrations in a study of healthy men and women from several racial and ethnic groups (24) so the current study aimed to determine the duration of Vit. D supplementation to reach an adequate or optimal Vit. D status and its effect on lipid profile.

**Methods**

In this longitudinal study 345 healthy women with different status of Vit. D levels were enrolled and followed up for one year. In this study, healthy women with age between 30-59 years were included. The exclusion criteria were a history of diseases including liver diseases and kidney, hypothyroidism and hyperparathyroidism, diabetes, pregnancy, identified intestinal diseases, and receiving Vit. D supplement at least 12 months before the initiation of the study.

The participants were selected via cluster sampling. At first, the name of all the health centers in Urmia City was listed and they clustered based on geographical status into four categories: north, south, west, and east. Then, several centers were selected from each cluster using the Excel randomly produced numbers. Finally, the required samples were selected randomly based on the ratio of total population in each health center.

The demographic data including age, education level, job, and sunlight exposure were collected via interviews with participants. Sun exposure data were collected through self-report by each participant and results were reported as days/week. Weight and height were measured and BMI was calculated by formula. Physical activity level was measured using a short standard International Physical Activity Questionnaire (IPAQ) (25) and results were reported as MET/min.

Each eligible participant received 50000 IU Vit. D 3 (cholecalciferol) was given orally once a month for 12 consecutive months. The serum Vit. D levels and lipid profile were measured at baseline (before intervention) and 3rd, 6th, and 12th months after the intervention. Serum 25-hydroxyvitamin D (25OHD) levels were measured using ELISA (IDS Ltd., Boldon, UK) and expressed as ng/mL. The lipid profile including total cholesterol, triglyceride, LDL-C, and HDL-C measured in mg/dl. According to the baseline data, the serum Vit. D levels of <20 ng/mL, 20-30 ng/mL, and >30 ng/mL are defined as deficiency, inadequacy, and adequacy, respectively 1(26).

This study was approved by the Ethics Committee of Urmia University of Medical Sciences and a written consent form was obtained from the participants.

**Statistical analysis**

Continuous variables were presented as mean ± standard deviation/standard error (SD/SE) and categorical variables were shown as n (%). The mean of age, BMI, physical activity, and lipid profiles (LDL-C, total cholesterol, and triglyceride) at baseline were compared using One-Way ANOVA. The chi-square test was used to compare the frequency of education level, job status, and sunlight exposure between three groups. Repeated measures were used for comparing mean changes of serum 25OHD levels level and lipid profiles during different times. Data analysis was performed using SPSS17 software and a p-value less than 0.05 was considered as significant level.

**Results**

In this study, 345 females were enrolled. Based on Vit. D level at baseline the participants were categorized into three groups: deficiency group (n=73), inadequate group (n=138), and adequate group (n=134).

The demographic and baseline characteristics of participants are shown in Table 1. None of the demographic variables showed statistically significant differences among the three groups. The mean of age was 36.93±9.92, 35.88±11.24, and 38.96±10.65 in deficiency, inadequate and adequate groups respectively. The differences between mean LDL-C levels and total cholesterol levels at baseline were significant. (p< 0.05). Also, the changes in HDL-C were significant, but in a downward trend.

Comparison of the serum Vit. D level in participants with different Vit. D status at baseline at different times showed that in all three groups the serum Vit. D level was increased significantly after one year of follow-up (p<0.05). In all participants the average of serum Vit. D level changes were 8 ng/mL at the end of the study. In three groups the Vit. D level was increased significantly. In the deficiency group, the mean changes of serum Vit. D level in the 6th and 12th months vs. the 3rd month was 4.08±0.85 and 10.01±1.02 ng/mL, respectively (p<0.001). In the inadequate group, the mean changes of serum Vit. D level in the 6th and 12th months vs. the 3rd month was 3.07±0.59 and 7.26±0.78 ng/mL, respectively (p=0.001). In the adequate group, the mean changes of serum Vit. D level in the 6th and 12th months vs. 3rd month was 2.02±0.88 and 6.44±1.005 ng/ml, respectively (p=0.001) (Table 2).

In Table 3 the effect of Vit. D supplementation on lipid profile has been compared at different times in each group. Results showed that at the end of the study (one year after follow-up) the lipid profile was improved. So, the mean changes of lipid profiles at the end of the study comparing the baseline were, -5.86±2.09, -7.22±1.43 and -6.17±1.72 (mg/dl) for LDL-C (p<0.05); -12.24±3.08, -13.64±3.21 and -17.81±2.94 (mg/dl) for total cholesterol (p<0.05) in deficiency, inadequate and adequate groups, respectively. For triglyceride, the mean changes were -13.24±5.78 and -15.85±7.49 (mg/dl) in deficiency and adequate groups, respectively (p<0.05). Although the triglyceride decreased in the inadequate group at the end of the study this difference was not significant (p=0.67).

**Discussion**

Recently, due to the high global prevalence of Vit. D deficiency (19), the use of its supplements has increased (5). Yet, the duration of Vit. D supplementation remained a controversial issue (12). So, the current study aimed to determine the Vit. D supplementation duration to reach an adequate or optimal Vit. D status and its effect on lipid profile.

The current study showed that 61.2 % of participants initially had a deficiency or insufficiency of vitamin D. Consistent with our finding, the epidemiological studies have shown that the prevalence of Vit. D deficiency was high in Iranian adults (26, 27). The results of the present study showed that receiving 50000 IU Vit. D_3_ monthly for 12 months increased the Vit. D level in participants with different serum Vit. D levels. In participants with Vit. D deficiency and inadequate level, the serum Vit. D level reached a sufficient level (>30 ng/mL), however, in an adequate group its level did not reach above 50 ng/mL.

In several studies the effect of different durations of supplementation with 50000 IU Vit. D has been investigated on different health outcomes (20-23, 28). In a study by Penckofer et al has shown that supplementation with 50000 IU weekly for 6 months increased the serum Vit. D; 25-OH from 20 ng/mL at baseline to above than 50 ng/mL (28). Another study has shown that a monthly supplementation with 80,000 IU Vit. D_3_ for 6 months corrected Vit. D insufficiency, without overdosing (29).

There are controversies about the healthy range of Vit. D levels, although the optimal level of Vit. D remains unclear; maintaining a serum 25(OH) D level of 40 - 60 ng/mL is recommended (30). Our findings showed that the serum Vit. D level did not reach above 50 ng/mL in subjects with the initial of Vit. D > 30 ng/mL during one-year supplementation. The need for Vit. D supplements in healthy individuals require careful clinical considerations to prevent undesirable long-term complications of supplements (31). In a study, the rates of hypervitaminosis were reported as 18.9% and 4.5% in weekly and biweekly users of 50,000 IU of Vit. D 3, respectively (21).

As much as Vit. D deficiency can be the cause of many diseases; its toxicity can also cause some complications. Vit. D overdosing can cause hypercalcemia, hypercalciuria, and over-deposition of minerals in soft tissues, and usually can be as a result of taking extremely high doses of Vit. D for a prolonged time (32,33). Malihi et al reported that one year or longer supplementation with a large daily, weekly, or monthly dose of Vit. D_2_/D_3_ did not significantly increase the risk of total adverse events or kidney stones, although there has been a trend towards increased hypercalcemia, and in some instances hypercalciuria (34). Other studies have shown that the level of safety of Vit. D_3_ supplementation is similar for doses of 400, 4000, and 10000 IU/day. Hypercalciuria was common and occurred more frequently with higher doses (35).

Our findings showed that the 50000 IU Vit. D_3_ supplementation improved the lipid profiles (LDL-C, total cholesterol, and triglyceride) in participants with different levels of Vit. D at baseline. At the end of this study, the lipid profiles decreased in three groups. Consistent with this study, in several previous studies such results have been earned and it has been shown that Vit. D_3_ supplementation has a beneficial effect on reducing lipid profiles (36-40).

It should be noted that several factors may further affect the amount of Vit. D required attaining a sufficient concentration (41). Intakes of Vit. D containing foods and geographical locations are the important and determinant factors for providing recommendations and the policies to achieve adequate Vit. D intake between the countries (42).

In summary, according to the prevalence Vit. D deficiency at the community level, Vit. D supplementation is necessary and more studies are needed to determine the appropriate timing and duration of supplementation in different states and countries. Nevertheless, to be able to provide updated knowledge regarding Vit. D intake and status in different population groups including infants, children, older adults, and pregnant women, it should be done wider studies for a safe duration of supplementation to improve Vit. D level.

**Conclusion**

Adequate Vit. D status is an important issue in regard the public health. The current study showed that the taking of 50000 IU Vit. D_3_ monthly for 12 months resulted in acquiring an adequate level in both deficiency and insufficient groups, without any risks of toxicity in the adequate group. Therefore, 50000 IU Vit. D_3_ supplementation monthly for one year can be safe and with beneficial effects on lipid profiles in healthy women and there is no elevating risk for Vit. D overdosing in such participants.

**Abbreviations**

**IPAQ**: International Physical Activity Questionnaire

**MET**: Metabolic Equivalent Task

**25(OH) D**: 25, hydroxy vitamin D3

**ELISA**: Enzyme-linked immunosorbent assay

LDL-C: Low-density lipoprotein Cholesterol

**BMI**: Body Mass Index

**SD:** Standard Deviation

**SE**: Standard Error

**Acknowledgments**

The authors would like to thank all respected experts in the health departments of Urmia University of Medical Sciences and other individuals who had sincere cooperation in conducting this study in the Ministry of Education in West Azerbaijan in Iran.

**Ethics approval and consent to participate**

The current study was conducted according to the guidelines in the Declaration of Helsinki, and procedures involving human subjects/patients were approved by the Urmia University of Medical Sciences (UMSU), Ethical Committee (No. IR.UMSU.REC.1398.002). Written informed consent was obtained from all participants before the study. The patients/participants provided their written informed consent to participate in this study.

**Availability of data and materials**

Please contact the author for data requests

**Authors' contributions**

DV was the study designer and program manager. SNS has participated in the data handling and analysis, the result, and full manuscript preparation. FK&FB has participated in executive management for data collection, sampling, and laboratory test work coordination. DV has participated in assessing the laboratory-used methods, statistical data analysis evaluation, scientific evaluation, and editing. All authors have seen and approved the final manuscript that has been submitted.

**Competing interests**

The authors declare that they have no competing interests.

**Consent for publication**

Not applicable.

**References**

1. Sizar O, Khare S, Goyal A, Givler A. Vitamin D deficiency. StatPearls [Internet]: StatPearls Publishing; 2022.

2. Zhang Y, Fang F, Tang J, Jia L, Feng Y, Xu P, et al. Association between vitamin D supplementation and mortality: systematic review and meta-analysis. Bmj. 2019;366-72.

3. Cantorna MT, Snyder L, Arora J. Vitamin A and vitamin D regulate the microbial complexity, barrier function, and the mucosal immune responses to ensure intestinal homeostasis. Critical reviews in biochemistry and molecular biology. 2019;54(2):184-92.

4. Fakhoury HM, Kvietys PR, AlKattan W, Al Anouti F, Elahi MA, Karras SN, et al. Vitamin D and intestinal homeostasis: Barrier, microbiota, and immune modulation. The Journal of steroid biochemistry and molecular biology. 2020;200:105663-70.

5. Amrein K, Scherkl M, Hoffmann M, Neuwersch-Sommeregger S, Köstenberger M, Tmava Berisha A, et al. Vitamin D deficiency 2.0: an update on the current status worldwide. European journal of clinical nutrition. 2020;74(11):1498-513.

6. Cui A, Xiao P, Ma Y, Fan Z, Zhou F, Zheng J, et al. Prevalence, trend, and predictor analyses of vitamin D deficiency in the US population, 2001–2018. Frontiers in Nutrition. 2022;9:2312-18.

7. Park J-H, Hong IY, Chung JW, Choi HS. Vitamin D status in South Korean population: Seven-year trend from the KNHANES. Medicine. 2018;97(26):1-7.

8. Nielsen NO, Jørgensen ME, Friis H, Melbye M, Soborg B, Jeppesen C, et al. Decrease in vitamin D status in the Greenlandic adult population from 1987–2010. PLoS One. 2014;9(12): 11294-300.

9. Szymczak-Pajor I, Śliwińska A. Analysis of association between vitamin D deficiency and insulin resistance. Nutrients. 2019;11(4):794-800.

10. Botelho J, Machado V, Proença L, Delgado AS, Mendes JJ. Vitamin D deficiency and oral health: a comprehensive review. Nutrients. 2020;12(5):1471-80.

11. Bouillon R, Manousaki D, Rosen C, Trajanoska K, Rivadeneira F, Richards JB. The health effects of vitamin D supplementation: Evidence from human studies. Nature Reviews Endocrinology. 2022;18(2):96-110.

12. Reid IR, Bolland MJ. Controversies in medicine: the role of calcium and vitamin D supplements in adults. Medical Journal of Australia. 2019;211(10):468-73.

13. Buttriss JL, Lanham-New SA, Steenson S, Levy L, Swan GE, Darling AL, et al. Implementation strategies for improving vitamin D status and increasing vitamin D intake in the UK: current controversies and future perspectives: proceedings of the 2nd Rank Prize Funds Forum on vitamin D. British Journal of Nutrition. 2022;127(10):1567-87.

14. Minisola S, Ferrone F, Danese V, Cecchetti V, Pepe J, Cipriani C, et al. Controversies surrounding vitamin D: focus on supplementation and cancer. International journal of environmental research and public health. 2019;16(2):189-96.

15. Stokes CS, Lammert F. Vitamin D supplementation: less controversy, more guidance needed. F1000Research. 2016;5:1-6.

16. Mendes M, Charlton K, Thakur S, Ribeiro H, Lanham-New SA. Future perspectives in addressing the global issue of vitamin D deficiency. Proceedings of the Nutrition Society. 2020;79(2):246-51.

17. Yadav A, Kumar J, Bhai VN. Vitamin D deficiency: definition matters! Indian Pediatrics. 2020;57(11):1083-90.

18. Gromova O, Doschanova A, Lokshin V, Tuletova A, Grebennikova G, Daniyarova L, et al. Vitamin D deficiency in Kazakhstan: cross-sectional study. The Journal of steroid biochemistry and molecular biology. 2020;199:105565-70.

19. Giustina A, Bouillon R, Binkley N, Sempos C, Adler RA, Bollerslev J, et al. Controversies in vitamin D: a statement from the third international conference. JBMR plus. 2020;4(12): 10417-25.

20. Bader DA, Abed A, Mohammad BA, Aljaberi A, Sundookah A, Habash M, et al. The Effect of Weekly 50,000 IU Vitamin D3 Supplements on the Serum Levels of Selected Cytokines Involved in Cytokine Storm: A Randomized Clinical Trial in Adults with Vitamin D Deficiency. Nutrients. 2023;15(5):1188-97.

21. Hashemipour S, Ghobadi A, Hadizadeh Khairkhah SMR, Ranjbaran M, Badri M, Ghafelehbashi SH, et al. Association of weekly or biweekly use of 50 000 IU vitamin D3 with hypervitaminosis D. British Journal of Clinical Pharmacology. 2022;88(7):3506-69.

22. Guan Y, Hao Y, Guan Y, Bu H, Wang H. The effect of vitamin D supplementation on rheumatoid arthritis patients: a systematic review and meta-analysis. Frontiers in Medicine. 2020;7:1-8.

23. AlAteeq MA, AlShail A, AlZahrani A, AlNafisah O, Masuadi E, Alshahrani A. Effect of Monthly and Bi-Monthly 50,000 International Units (IU) Maintenance Therapy With Vitamin D3 on Serum Level of 25-Hydroxyvitamin D in Adults: A Randomized Controlled Trial. Cureus. 2021;13(3):5-12.

24. Grimnes G, Figenschau Y, Almås B, Jorde R. Vitamin D, insulin secretion, sensitivity, and lipids results from a case-control study and a randomized controlled trial using hyperglycemic clamp technique. Diabetes. 2011;60(11):2748-57.

25. Craig CL, Marshall AL, Sjöström M, Bauman AE, Booth ML, Ainsworth BE, et al. International physical activity questionnaire: 12-country reliability and validity. Medicine & science in sports & exercise. 2003;35(8):1381-95.

26. Cashman KD. Vitamin D deficiency: defining, prevalence, causes, and strategies of addressing. Calcified tissue international. 2020;106(1):14-29.

27. Tabrizi R, Moosazadeh M, Akbari M, Dabbaghmanesh MH, Mohamadkhani M, Asemi Z, et al. High prevalence of vitamin D deficiency among Iranian population: a systematic review and meta-analysis. Iranian journal of medical sciences. 2018;43(2):125-34.

28. Vatandost S, Jahani M, Afshari A, Amiri MR, Heidarimoghadam R, Mohammadi Y. Prevalence of vitamin D deficiency in Iran: a systematic review and meta-analysis. Nutrition and health. 2018;24(4):269-78.

29. Penckofer S, Ridosh M, Adams W, Grzesiak M, Woo J, Byrn M, et al. Vitamin D supplementation for the treatment of depressive symptoms in women with type 2 diabetes: a randomized clinical trial. Journal of Diabetes Research. 2022;2022:1-10.

30. Tournier H, Tran N, Dray N, Brusset M, Rander B, Millot X, et al. Supplementation with 80,000 IU vitamin D3/month between November and April corrects vitamin D insufficiency without overdosing: Effect on serum 25-hydroxyvitamin D serum concentrations. La Presse Médicale. 2017;46(3): 69-75.

31. MOC C. Vitamin D supplementation: Pearls for practicing clinicians. Cleveland Clinic Journal of Medicine. 2022;89(3):155-63.

32. Razzaque MS. Can adverse effects of excessive vitamin D supplementation occur without developing hypervitaminosis D? The Journal of steroid biochemistry and molecular biology. 2018;180:81-6.

33. Pludowski P, Holick MF, Grant WB, Konstantynowicz J, Mascarenhas MR, Haq A, et al. Vitamin D supplementation guidelines. The Journal of steroid biochemistry and molecular biology. 2018;175:125-35.

34. Pérez-Barrios C, Hernández-Álvarez E, Blanco–Navarro I, Pérez-Sacristán B, Granado-Lorencio F. Prevalence of hypercalcemia related to hypervitaminosis D in clinical practice. Clinical Nutrition. 2016;35(6):1354-8.

35. Malihi Z, Wu Z, Lawes CM, Scragg R. Adverse events from large dose vitamin D supplementation taken for one year or longer. The Journal of steroid biochemistry and molecular biology. 2019;188:29-37.

36. Billington EO, Burt LA, Rose MS, Davison EM, Gaudet S, Kan M, et al. Safety of high-dose vitamin D supplementation: secondary analysis of a randomized controlled trial. The Journal of Clinical Endocrinology & Metabolism. 2020;105(4):1261-73.

37. Dibaba DT. Effect of vitamin D supplementation on serum lipid profiles: a systematic review and meta-analysis. Nutrition reviews. 2019;77(12):890-902.

38. Challoumas D. Vitamin D supplementation and lipid profile: what does the best available evidence show? Atherosclerosis. 2014;235(1):130-9.

39. Barzegari M, Sarbakhsh P, Mobasseri M, Noshad H, Esfandiari A, Khodadadi B, et al. The effects of vitamin D supplementation on lipid profiles and oxidative indices among diabetic nephropathy patients with marginal vitamin D status. Diabetes & Metabolic Syndrome: Clinical Research & Reviews. 2019;13(1):542-7.

40. Khosravi ZS, Kafeshani M, Tavasoli P, Zadeh AH, Entezari MH. Effect of Vitamin D supplementation on weight loss, glycemic indices, and lipid profile in obese and overweight women: A clinical trial study. International journal of preventive medicine. 2018;9:1-9.

41. Ramasamy I. Vitamin D metabolism and guidelines for vitamin D supplementation. The Clinical Biochemist Reviews. 2020;41(3):103-108.

42. Itkonen ST, Andersen R, Björk AK, Brugård Konde Å, Eneroth H, Erkkola M, et al. Vitamin D status and current policies to achieve adequate vitamin D intake in the Nordic countries. Scandinavian journal of public health. 2021;49(6):616-27.

**Table 1- The demographic characteristics of participants**

| Variables |  | deficiency group  (< 20 ng/mL) | inadequate group  (20-30 ng/mL) | adequate group  (>30 ng/mL) | p-value |
| --- | --- | --- | --- | --- | --- |
|  |  | n=73 | n=138 | n=134 |  |
| age |  | 36.93±9.92^*^ | 35.88±11.24 | 38.96±10.65 | 0.06^¶^ |
| BMI |  | 27.71±13.92 | 27.96±11.42 | 26.68±5.31 | 0.58^¶^ |
| Physical activity (MET/Min) |  | 2430.24±314.09 | 2272.69±215.06 | 2511.94±244.35 | 0.76^¶^ |
| Job, n (%) | Un-employ | 42 (57.5) | 93 (67.4) | 85 (63.9) | 0.37^¶¶^ |
|  | employed | 31 (42.5) | 45 (32.6) | 48 (36.1) |  |
| Education, n(%) | Low level | 11 (15.1) | 40 (29.2) | 34 (26.0) | 0.07^¶¶^ |
|  | High level | 62 (84.9) | 97 (70.8) | 97 (74.0) |  |
| sunlight exposure, n (%), < 2 / > 2 days | baseline | 63 (95.5)/ 3(4.5) | 114 (95) / 6 (5) | 10 (95.5) / 5 (4.5) | 0.98^¶¶^ |
|  | 3^th^ month | 12 (16.4) / 61 (83.6) | 47 (34.1) / 91 (65.9) | 48 (35.8) / 86 (64.2) | 0.01^¶¶^ |
|  | 6^th^ month | 59 (80.8) / 14 (19.2) | 121 (87.7.1) / 17 (12.3) | 125 (93.3) / 9 (6.7) | 0.03^¶¶^ |
|  | 12^th^ month | 12 (16.4) / 61 (83.6) | 34 (24.6) / 104 (75.4) | 29 (21.6) / 105 (78.4) | 0.39^¶¶^ |
| LDL-C (mg/dl) |  | 94.66±24.6 | 96.26±23.98 | 104.28±24.98 | 0.006^¶^ |
| Total Cholesterol (mg/dl) |  | 179.14±37.71 | 179.25±43.12 | 194.22±38.42 | 0.004^¶^ |
| Triglyceride (mg/dl)  HDL-C |  | 157.19±78.7  49.09±9.25 | 152.43±87.33  49.85±15.02 | 152.16±74.03  52.49±10.90 | 0.89^¶^  0.07 |

¶: Comparing mean between three groups using One-Way ANOVA.

¶¶: Comparing frequency between three groups using Chi-square test.

*: data are presented as Mean±SD.

**Table 2- Comparing the vitamin D serum level between different times in participants with different vitamin D status at baseline**

| Times | deficiency group  (< 20 ng/mL) | inadequate group  (20-30 ng/mL) | adequate group  (>30 ng/mL) |
| --- | --- | --- | --- |
| 3^th^ month | 22.82±9.02^*^ | 30.59±14.36 | 35.07±13.68 |
| 6^th^ month | 26.9±9.36 | 33.67±13.24 | 37.10±13.15 |
| 12^th^ month | 32.84±10.2 | 37.86±14.29 | 41.51±14.5 |
| Mean difference 6^th^ month vs. 3^th^ month | 4.08±0.85^**^ | 3.07±0.59 | 2.02±0.88 |
| Mean difference 12^th^ month vs. 3^th^ month | 10.01±1.02 | 7.26±0.78 | 6.44±1.005 |
| p-value^¶^ | <0.001 | 0.001 | 0.001 |

¶: Comparing mean between three times using repeated measures (adjusted for sunlight exposure).

*: data are presented as Mean±SD.

**: data are presented as Mean±SE.

**Table 3- The effect of vitamin D supplementation on lipid profile in participants with different vitamin D status at baseline**

| variable | Times | deficiency group  (< 20 ng/mL) | inadequate group  (20-30 ng/mL) | adequate group  (>30 ng/mL) |
| --- | --- | --- | --- | --- |
| LDL_C (mg/dl) | baseline | 94.66±24.61^*^ | 96.26±23.98 | 104.28±24.98^*^ |
|  | 3^th^ month | 90.71±25.92 | 93.77±25.61 | 100.32±24.06 |
|  | 6^th^ month | 89.96±24.61 | 92.06±23.05 | 100.54±22.25 |
|  | 12^th^ month | 88.82±24.54 | 89.04±22.59 | 98.11±21.68 |
| p-value^¶^ |  | 0.014 | <0.001 | 0.002 |
| Total Cholesterol(mg/dl) | baseline | 179.14±37.71 | 179.25±43.12 | 194.22±38.42 |
|  | 3^th^ month | 166.09±39.91 | 169.85±35.68 | 179.8±37.55 |
|  | 6^th^ month | 165.2±39.53 | 166.0±34.29 | 179.14±36.45 |
|  | 12^th^ month | 166.89±89 | 165.38.32 | 176.4±37.66 |
| p-value |  | <0.001 | <0.001 | <0.001 |
| Triglyceride(mg/dl) | baseline | 157.19±78.74 | 152.43±87.33 | 168.01±97.8 |
|  | 3^th^ month | 146.7±71.62 | 151.49±92.21 | 166.16±65.8 |
|  | 6^th^ month | 143.38±62.94 | 155.81±82.24 | 157.79±68.45 |
|  | 12^th^ month | 143.94±65.86 | 149.04±72.65 | 152.16±74.03 |
| p-value |  | <0.001 | 0.67 | 0.038 |
| HDL-C | baseline | 49.09±9.25^*^ | 49.85±15.02 | 52.49±10.90^*^ |
|  | 3^th^ month | 48.95±11.60 | 49.78±15.03 | 49.50±10.00 |
|  | 6^th^ month | 48.67±11.57 | 47.62±15.81 | 49.37±10.75 |
|  | 12^th^ month | 48.09±12.41 | 45.88±16.28 | 47.91±11.23 |
| p-value |  | 0.77 | 0.001 | <0.001 |

¶: Comparing mean between four times using repeated measures (adjusted for sunlight exposure).

*: data are presented as Mean±SD.


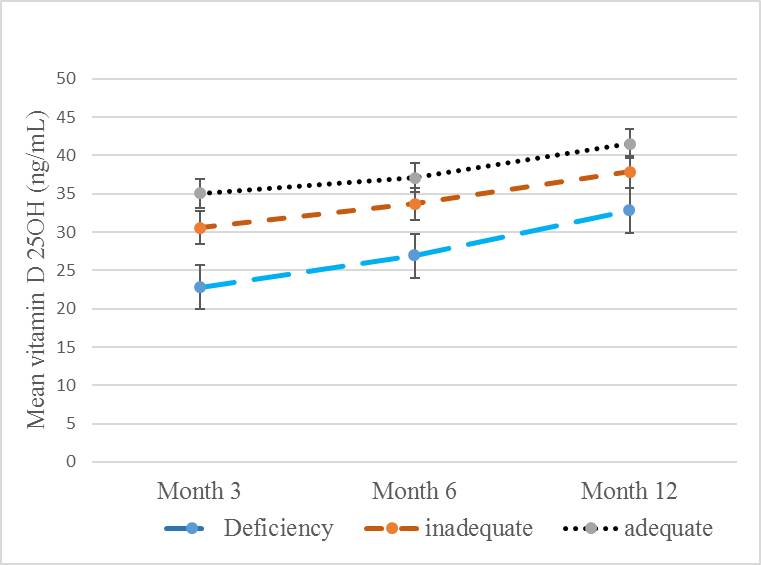


**Fig 1- Mean serum vitamin D (25 OH D) between different groups during intervention time**
